# Identity Formation in Individuals between 16 and 25 Years Old with Borderline Personality Disorder

**DOI:** 10.3390/jcm13113221

**Published:** 2024-05-30

**Authors:** Anaïs Mungo, Marie Delhaye, Camille Blondiau, Matthieu Hein

**Affiliations:** 1 CH Le Domaine-ULB, Department of Psychiatry, Université Libre de Bruxelles, 1420 Braine l’Alleud, Belgium; 2 CHU HELORA, Department of Psychiatry, Hôpital de La Louvière-Site Jolimont, 7100 La Louvière, Belgium; mariemd.delhaye@gmail.com; 3 HUB—Site Anderlecht, Department of Baby, Child, Adolescent and Young Adult Psychiatry, Université Libre de Bruxelles, 1070 Bruxelles, Belgium; camille.blondiau@hubruxelles.be; 4 HUB—Site Anderlecht, Department of Psychiatry and Sleep Laboratory, Université Libre de Bruxelles, 1050 Brussels, Belgium; matthieu.hein@ulb.be

**Keywords:** borderline personality disorder, identity, young adult, DIDS, development, impulsive behaviors

## Abstract

**Background/Objectives**: Identity disruption is a key feature of borderline personality disorder (BPD), characterized by disturbances in self-image. This study aimed to use the Dimensions of Identity Development Scale (DIDS) in a population aged 16–25, to assess differences in identity status and correlations with BPD features as well as whether a correlation exists between the BPD features, the scores obtained on the DIDS and the scores of the different dimensions of this disorder. **Methods:** We analyzed data from 132 individuals: 44 with BPD using the Diagnostic Interview for Borderline—Revised (DIB-R). Statistical analyses included quantile regression to determine the differences in the DIDS after adjusting for confounding factors identified during group comparisons and Spearman correlation between the DIDS, the BPD features and the DIB-R. **Results:** Results indicated significantly lower DIDS scores in the BPD group, particularly in commitment making, exploration breadth (EB), identity with commitment (IM) and ruminative exploration (RE). After adjusting, only EB differs significantly between the two groups. All dimensions of the DIDS except for the exploration in depth (ED) are correlated with BPD features. Significant correlations could be demonstrated between cognitive dimension and ED, between the total DIDS and the number of suicide attempt (SA) and between the IM and the number of SA. **Conclusions:** Our clinical sample showed distinct identity formation compared to controls, with a lower EB associated with BPD. RE correlated with BPD, suggesting that the individuals engage in repetitive exploratory processes. SA was negatively associated with overall identity development and commitment, indicating impulsive behaviors in BPD intersect with identity struggles.

## 1. Introduction

Borderline personality disorder (BPD) is a common disorder with a prevalence of between 0.9% and 3% in the adolescent and young adult population [1,2]. Identity disturbance is one of nine criteria diagnostics in the DSM-5 nomenclature system that characterizes this disorder. According to the DSM-5 dimensional model, in BPD, the disturbance of identity manifests as a markedly impoverished, underdeveloped, or unstable self-image, and is associated with excessive self-criticism, chronic feelings of emptiness, and dissociative states, particularly when experiencing stress [3].

Identity disturbance is complex and is one of the main features of BPD [4,5]. BPD is one of the most prototypical disorders among identity-related problems [6]. Zanarini conceptualizes borderline identity as showing a tendency to overstate a sense of worthlessness and a negative self-image with limited positive self-regard [4]. Kernberg and colleagues view it as diffuse, meaning that individuals with borderline disorder fail to integrate positive and negative aspects of self and others, resulting in representations of themselves and others being presented in a fragmented or unstructured manner. According to Kernberg, this diffusion of identity is reflected by a difficulty in engaging in interpersonal relationships and interferes with the construction of personal goals and interests [4,7].

Insecure attachment, characteristic in the development of this personality disorder, and deficits in the development of trust inhibit the establishment of a stable and mature identity [8]. There is indeed a strong association between “preoccupied” attachment (anxious in children) or “insecure” and borderline disorder [9,10]. Preoccupied individuals have a negative view of themselves and a positive view of others, leading to a drop in self-esteem. They tend to idealize helpers and often depend on others. Feelings of anger and distress are intense, especially in situations that evoke loss or abandonment [10].

The sense of confusion about self and others that characterizes individuals with attachment disorganization closely resembles the identity diffusion typical of individuals with BPD [6]. However, identity disturbance is not exclusive to BPD. Problems with identity are also linked with mood disorders, eating disorders, substance abuse, suicidality, and schizophrenia. Individuals with severe psychopathology show signs of disrupted identity formation early in development [11].

Borderline personality disorder is a complex and multifactorial condition. Currently, there is no consensus on how this pathology develops [1]. However, it seems that gene–environment interactions play a role in its development. This suggests that there may be a genotype that increases the risk of developing borderline personality disorder (the phenotype) if the environment predisposes the individual, particularly through epigenetic mechanisms [1,12]. Frontolimbic dysfunctions appear to be involved in borderline disorder and its associated characteristics, as indicated by the meta-analysis by Winsper et al. [12]. Specifically, these dysfunctions include the anterior cingulate cortex, the dorsolateral prefrontal cortex, the amygdala, and the hippocampus, with reductions in gray matter volume and alterations in white matter. Additionally, structural abnormalities such as reduced volumes in the amygdala, hippocampus, orbitofrontal cortex, and cingulate cortex have been observed [1,13]. There is also evidence of hyperreactivity in the hypothalamic-pituitary–adrenal axis [13]. Phenotypically, these dysfunctions contribute to emotional regulation problems and impulsive behavior in individuals with borderline personality disorder [1,13].

Human identity is constructed based on biological and behavioral processes and through interactions with others, particularly with the family, and is under the influence of culture and societal rules [8,11]. It develops mainly during adolescence and in the first years of adulthood, the period in which BPD is generally diagnosed [8].

Clinically, identity disturbance corresponds to difficulties with describing personal characteristics and those of others, as well as problems with developing a sense of self with stable beliefs, interests, and life goals [14]. Such individuals experience rapid changes in how they view themselves and others; they also exhibit feelings of inner emptiness [15]. However, it is important to distinguish the pathological identity diffusion in BPD from the less severe and transient type in adolescence. An adolescent or a young adult with momentary identity problems has a fundamental sense of self and a structural basis for forming an identity [6]. Unlike individuals with BPD, they usually have an idea of their problems and the conflicting nature they are experiencing, and are able to describe their personality [15].

Several studies have focused on describing identity disturbances in adolescents with BPD, with findings suggesting that these disturbances are actually characteristic of BPD and can be distinguished from those seen in other personality disorders [16,17,18,19]. Adolescents and young adults reporting higher levels of identity confusion are more likely to exhibit low self-esteem, feelings of emptiness, dissociation and an unstable self-concept [15]. In a non-clinical population, Campbell et al. found that identity confusion and disturbance were associated with more borderline symptoms [20].

Despite the prevalence of identity disruption in BPD, our understanding of identity diffusion from a developmental point of view is insufficient due to a paucity of relevant studies [8]. It is further limited by the low quality or inappropriateness of available assessment tools and research methods. Another challenge is that identity formation cannot be directly observed by external evaluators, as it is largely characterized by subjective distress and private experiences [5]. The current scarcity of studies is perhaps the result of these difficulties along with operationalizing and capturing the construction of self-image in clinical research [21,22]. A few studies with adult clinical samples have shown that difficulty in identity development is associated with symptoms of BPD [20,23].

### 1.1. Definition of Identity

The term “identity” encompasses several notions [24]. According to Erikson’s conceptualization, identity is the result of a dynamic process between the individual and their life context, where the individual attempts to reconcile their needs and preferences with those of society. If this process is successful, establishing a personal identity allows the individual to experience a sense of well-being, security, and stability, and to move forward in life with purpose and direction [25]. This phenomenon is considered to be universal. Identity has been described as “an organization of self-understanding that defines one’s place in the world” [24] (p. 5) and refers to the values and beliefs that an individual adopts and wishes to defend [24,25].

### 1.2. The Development of Identity

#### 1.2.1. Marcia’s Approach

According to Marcia, two independent dimensions contribute to identity development, particularly among adolescents: (1) exploration, which seeks to explore different identity alternatives in several areas of life, and (2) commitment, which represents adherence to a specific set of values and beliefs [26,27].

Crossing these two dimensions generates four identity statuses: [28,29]

- Realization (acquisition) of identity: This is the most successful and mature status, that of a person who is no longer in search of identity but who has defined the identity elements to which they adhere. Individuals went through an exploration phase before making firm commitments.
- Identity moratorium: This corresponds to a state of exploration and a lack of commitment by individuals who can still define several possible alternatives when making choices. Individuals are currently in a state of exploration but have not made strong commitments.
- Identity foreclosure: This is a state of commitment to several goals without any exploration and corresponds to individuals who are unsure and who still identify with parental models. Individuals have adopted commitments without prior exploration.
- Diffusion of identity: This is associated with a lack of exploration and commitment. Behaviors are unsuitable, even risky. It is a type of absence of basic identity structure. Individuals did not experience a proactive exploratory period and did not make firm commitments.

Individuals with a consolidated identity are better able to adapt to different contexts, modulate their behavior without losing a fundamental sense of internal coherence and stability, and act in a relatively coherent and predictable way. A diffuse sense of identity compromises the individual as an autonomous subject and actor [8].

#### 1.2.2. Luyckx’s Approach

In an extension of the theory proposed by Marcia, Luyckx and his collaborators formulated a model called “in double-cycle” that distinguishes five identity processes, including three processes of exploration and two processes relating to commitments [27,30].

In Luyckx’s theory, exploration and engagement are each divided into two dimensions: (1) exploration in breadth, referring to the balancing of various identity alternatives in the absence of actual engagement, and (2) in-depth, defined as the analysis of commitments already made [27,28]. A third form of exploration, “ruminative”, can be problematic when the individual remains stuck in a repetitive exploratory process marked by hesitation and indecision [27,30]. Commitment includes “commitment making”, as defined by Marcia, and “identification with commitments”, referring to a strong sense of belonging to commitments already made [27,28]. These four processes are implemented successively in identity formation. Thus, individuals must first consider several alternatives (exploration in breadth) and then commit to one (commitment making). The person continues to evaluate the selected alternative—in-depth exploration. They can then identify themselves or not with this choice—identification with the commitment [28].

This “double-cycle” model led to the construction of the “dimensions of identity development scale” (DIDS) measurement [27,31]. Individuals with identity achievement status typically score high on both dimensions of engagement (i.e., making engagement and identifying with engagement), moderate-to-high scores for breadth and depth exploration, and low scores in ruminative exploration. Similarly, people with foreclosure status score high on both dimensions of engagement but low on all dimensions of exploration. Individuals within the moratorium, considered as representing a transitory “crisis”, obtain an intermediate score low on the two dimensions of engagement but high on all the dimensions of exploration. Those within identity diffusion score much lower in exploration, especially the ruminative type [32].

### 1.3. Study Aims

This study has three objectives: (1) To test the “dimensions of identity development scale” (DIDS) for the first time in a clinical population with BPD aged 16–25 years. (2) Assess the differences in identity status between patients with BPD and a control population of the same age group. (3) Assess whether a correlation exists between the scores obtained on the DIDS and the scores of the different dimensions of the “Diagnostic Interview for Borderline—Revised” (DIB-R). The DIB-R quantitatively and qualitatively assesses the dimensions of BPD: affects, cognition, impulsive behaviors, and interpersonal relationships [33].

## 2. Materials and Methods

### 2.1. Population

We recruited both clinical and non-clinical populations. Clinical population was recruited among patients attending outpatient or inpatient care in the Adolescent Psychiatry Department and the Adult Psychiatry Department of an Academic General Hospital in the Brussels urban area.

The inclusion criteria for the clinical population studied were as follows:

- Presence of BPD, according to DSM-5 criteria, verified by the treating psychiatrist and confirmed by one investigator with substantial clinical experience of BPD, and through the semi-structured Diagnostic Interview for Borderline—Revised (DIB-R).
- Aged 16–25 years old.
- Understand and speak fluent French.

The exclusion criteria were as follows:

- Refusal to participate in the study;
- Inability to respond to assessments;
- Intellectual disability;
- A psychotic disorder (e.g., schizophrenia or pervasive developmental disorder);
- A serious somatic pathology (e.g., cancer, heart failure, renal failure, or central neurological disease) that is progressive or likely to jeopardize the vital prognosis in the short term.

Concerning the non-clinical population, we recruited both secondary school and university students. For the controlled individuals, the inclusion criteria were ages between 16 and 25 years old and to be fluent in French. The exclusion criteria were the following:

- Refusal to participate in the study;
- Inability to respond to assessments;
- A psychiatric disorder or a psychiatric history;
- A serious somatic pathology (e.g., cancer, heart failure, renal failure, or central neurological disease) that is progressive or likely to jeopardize the vital prognosis in the short term.

### 2.2. Method

The study procedure was carefully explained, and written informed consent was obtained. For minors, the consent of a parent or legal guardian was mandatory. Study questionnaires were administered in French.

Only patients with a diagnosis of BPD according to the DSM-5 criteria were recruited and confirmed by prior assessment using the DIB-R. The DIB-R quantitatively and qualitatively assesses the dimensions of BPD: affects, cognition, impulsive behaviors, and interpersonal relationships. Each dimension includes items rated 0 (negative response), 1 (probable), or 2 (positive). The items are grouped into summary statements and are then transformed, according to precise instructions, into graduated scores. The final DIB-R score ranges from 0 to 10. A DIB-R score of more than 8 confirms the diagnosis of PD but is acceptable from 7 onwards [33]. The DIB-R’s baseline test–retest reliability, interrater reliability, follow-up interrater reliability, and follow-up longitudinal reliability of borderline symptoms are all excellent with kappa values > 0.75 [33].

Construction of identity was assessed using the French adaptation of the DIDS; it was validated in French by Zimmerman et al. in 2015 [31]. The construction of identity was assessed using the DIDS. This measure gauges future plans and life paths on five identity dimensions: exploration in breadth, in-depth exploration, engagement, identification with engagement, and ruminative exploration. The scale has 25 items, with 5 for each dimension. Each item was rated on a Likert scale of 1 (completely disagree) to 5 (completely agree) [27]. There is no cut-off score for the scale or for the different dimensions. The internal reliability of the subscales was excellent (α > 0.8), except for the exploration in depth dimension (α < 0.7) [31]. The DIDS has previously produced consistent results in studies conducted with Belgian–Dutch, German, Turkish, Filipino, American, Swiss, and French adolescents from the general population [32]. The scoring of the DIDS is organized as follows [31]:

- Items 1–5 assess commitment making, e.g., “I have decided on the direction I want to follow in my life”;
- Items 6–10 assess exploration in breadth, e.g., “I think actively about different directions I might take in my life”;
- Items 11–15 assess ruminative exploration, e.g., “I keep wondering, which direction my life has to take”;
- Items 16–20 assess identification with commitment, e.g., “I sense that the direction I want to take in my life will really suit me”;
- Items 21–25 assess exploration in depth, e.g., “regularly talk with other people about the plans for the future I have made for myself”.

We also collected self-reported sociodemographic data regarding gender, age, marital status, having children, having living parents, number of siblings, place in sibling order, student status, education level, somatic history, surgical history, psychiatric history, presence of psychiatric comorbidities, alcohol use, drug use, family psychiatric history, history and number of suicide attempts, psychiatric treatment, and criminal history.

### 2.3. Statistical Analyses

Statistical analyses were performed using version 14 of the Stata software.

Two groups were formed: the control group of normative individuals and the study group of subjects with BPD.

Categorical data were described by percentages and numbers while continuous variables were described by their median and its P25–P75.

Data were analyzed by the Wilcoxon test for continuous variables and by the Chi^2^ test and Fisher’s exact test for dichotomous variables.

In a second step, the groups were compared with the Mann–Whitney–Wilcoxon test regarding the different scores on the DIDS.

A quantile regression test was performed to assess whether the results for the different scores on the DIDS remained significant after adjusting for potential confounding factors identified during group comparisons.

Finally, Spearman correlation was used to test between the scores of the different dimensions of the DIDS and the presence of the BPD features in the whole sample. We also conducted Spearman correlations between the different dimensions of DIB-R, and DIDS was carried out as well as for the number of suicide attempts.

A *p*-value of 0.05 was considered significant.

### 2.4. Ethics Statement

This research protocol was approved by the Erasme-ULB Hospital Faculty Ethics Committee, Brussels, Belgium (reference: P2020/111). Written informed consent to participate in this study was provided by the participants, if they were above 18 years old, or by their legal guardian/next of kin if they were below 18 years old.

## 3. Results

### 3.1. Demographic Data

In total, 132 participants aged 16–25 (81% female) were included for study analysis: 44 participants with BPD (93.2% female) and 88 control ones (75% female). Median age was 18 years [17,18,19,20,21,22]. Regarding the level of education, 29.6% of the non-clinical population have a high school diploma and 31.8% were in secondary school, while for the BPD population, 4.5% have a high school diploma and 25.0% have a secondary diploma.

As shown in Table 1, the following characteristics were significantly more frequent among young people with BPD: (1) female (93.2% vs. 75%, *p* = 0.012), (2) deceased parents (11.4% vs. 2.3%, *p* = 0.028), (3) the eldest sibling (38.6% vs. 21.6%) and the middle sibling (25% vs. 12.5%) (*p* = 0.001), (4) a psychiatric history (47.7% vs. 0.0%, *p* < 0.001), (5) psychiatric comorbidities (61.4% vs. 0.0%, *p* < 0.001), (6) smoking (38.6% vs. 4.5%, *p* < 0.001), (7) an occasional alcohol consumption (75% vs. 53.4%, *p* = 0.043), (8) drug consumption (50% vs. 5.7%, *p* < 0.001), (9) a psychiatric family history (86.4% vs. 21.6%, *p* < 0.001), (10) a history of attempted suicide (75% vs. 12.5%, *p* < 0.001), (11) past psychological follow-up (79.5% vs. 17%, *p* < 0.001), (12) in treatment (72.7% vs. 0%, *p* < 0.001), and (13) no high school diploma (95.5% vs. 70.4%, *p* = 0.001).

The number of suicide attempts differed significantly between the two groups (*p* < 0.001). The median number of suicide attempts among individuals with borderline is two, and zero in the control group.

### 3.2. Comparison of DIDS Scores

Table 1 shows that compared to controls, the total score on the DIDS is significantly lower in the population with BPD (92.0 [86.0–99.0] vs. 81.0 [63.0–93.0], *p* < 0.001). It also differs significantly regarding the dimensions of commitment making (21.0 [18.0–24.0] vs. 16.0 [10.0–20.0], *p* < 0.001), exploration in breadth (21.0 [19.0–23.0] vs. 17.0 [13.0–20.0], *p* < 0.001), ruminative exploration (14.0 [10.0–19.0] vs. 17.0 [13.0–22.0], *p* = 0.044), and the level of identification with commitment (19.0 [16.0–23.0] vs. 12.0 [8.0–17.0], *p* < 0.001). Based on the medians of the scores on the DIDS, the control group seems to be in a status of identity realization, while the BPD group is in a status categorized as moratorium [32].

### 3.3. Quantile Regression Test

After adjusting for the confounding factors—gender, living parents, place among siblings, psychiatric history, psychiatric comorbidities, smoking, alcohol use, drug use, family psychiatric history, history of attempted suicide, a past follow-up, taking psychotropic treatment, and education level—only exploration in breadth differs significantly between the two groups (*p* < 0.05) (Table 2).

### 3.4. Correlation Analyses

Table 3 displays the correlations between identity dimensions and the presence of BPD features in the entire sample. Presence of BPD features were positively correlated with ruminative exploration (Spearman r = 0.177, *p* < 0.05) and negatively with commitment making (Spearman r = −0.395, *p* < 0.05), exploration in breadth (Spearman r = −0.467, *p* < 0.05), identification with commitment (Spearman r = −0.511, *p* < 0.05), and the total DIDS (Spearman r = −0.425, *p* < 0.05).

Table 4 illustrates the correlations between DIDS dimensions and BPD dimensions according to the DIBR (affects, cognition, impulsivity, and interpersonal relationships), along with the number of suicide attempts in the young adult sample with BPD. Significant correlations could be demonstrated between cognitive dimension and exploration in depth (Spearman r = 0.300, *p* < 0.05), between the total DIDS and the number of suicide attempts (Spearman r = −0.351, *p* < 0.05), and between the identification with commitment and the number of suicide attempts (Spearman r = −0.380, *p* < 0.05).

## 4. Discussion

The main objective of this study was to test the DIDS in a population of young adults with BPD aged 16 and 25 and to assess differences in the construction of identity in this population. The results should be placed within a developmental context. Indeed, the age of 16 to 25 is a period of transition to adulthood. This is a time of vulnerability. It is a period of great changes at the physiological level (cerebral maturation), psychological (identity development, sense of belonging, etc.), and at the social level (entry into professional life, end of secondary school, empowerment, integration).

Concerning demographics, the differences observed between the two groups confirm the data in the literature. Women are more often diagnosed with BPD, and a ratio of 3 women for 1 man is reported in the literature. However, this female predominance is probably linked to many biases and can be explained by different reasons, including a greater demand for care by women than men [34]. Patients with BPD have more psychiatric comorbidities, many different follow-ups as well as more psychiatric antecedents [35,36]. Compared with other mental disorders, the frequency of comorbidities would be significantly higher in these young patients. They are at higher risk for substance abuse (smoking, alcohol, and drugs) and suicide attempts. Adolescents with borderline disorder also present more difficulties at school. The presence of psychiatric disorder in the family—more particularly a substance use disorder in the father and a BPD in the mother—also favor the emergence of BPD in adolescence [1,2]. A history of childhood trauma is often associated with BPD, and in the study by Tate et al., the death of a parent was associated with BPD. Contrary to the literature, in our study, we do not find any difference between the two groups regarding somatic pathologies. This is probably due to the young age of our sample. BPD usually presents a high risk of somatic pathologies [36].

Regarding the different scores on the DIDS, commitment making, exploration in breadth, and identification with commitment are significantly lower for young adults with BPD than for the control group. Jorgensen found that individuals diagnosed with BPD exhibited notably lower scores in identity commitment compared to adults from the general community, and identification with commitment usually predicts better functioning in individuals [23,32]. In our sample, this is the lowest score, while the other two scores are high on average. BPD is associated with significant deficits in psychosocial functioning [1], which could be related to the deficit in identification with commitment. It would have been interesting to evaluate a link between this score and an overall functioning score. Moreover, low commitment is often associated with internalizing symptoms such as depression and anxiety [20,37]. While more comorbidities were observed in our sample with BPD, we did not specifically assess the presence of anxiety or depression. Ruminative exploration was also significantly higher in this group, suggesting a tendency to remain stuck in a repetitive exploratory process.

According to Mannerström, identity construction is influenced by the environment in which the individual develops, particularly their education, income, and life context [32]. The environment in which the young person develops can therefore influence their DIDS scores [32]. We conducted a quantile regression test to assess whether the differences of the DIDS scores between the two groups were linked to the confounding sociodemographic factors found during our comparison analysis. These confounding factors were gender, living parents, place among siblings, psychiatric history, psychiatric comorbidities, smoking, alcohol use, drug use, family psychiatric history, history of attempted suicide, a past follow-up, taking psychotropic treatment, and education level. Only exploration in breadth remained significantly different after controlling for these factors. This dimension is likely related to intrinsic aspects of borderline personality disorder and is independent of external factors. The differences between the two groups did not explain the difference in the ability of exploration in breath. Exploration in breadth is the ability to evaluate alternatives in identity development and could pose the most difficulty of all dimensions for young adults with BPD [28]. Our clinical population in this study have more difficulty to evaluate different alternatives in their life projects. Contrary to what one might think, alternatives would not be influenced by the familial environment, school, psychiatric comorbidities, and other confounding factors. According to Zanarini, individuals with BPD have a vacillating and unstable self-image that does not allow them to evaluate alternatives. Indeed, the self and, more particularly, the sense of self-coherence is a product of mentalizing capacities. Individuals with BPD seem to have difficulty of mentalization and more particularly, in intense emotional states. This therefore does not allow them to balance various identity alternatives that characterize exploration in breadth [6]. This negative identity would, nevertheless, become less severe over time [7]. A future goal is to assess whether exploration in breadth in individuals with BPD also improves over time and with a better self-image. Moreover, identity exploration involves periods of solitude that allow you to discover yourself and to deepen different identity options. Consequently, avoiding others and preferring to be alone could sometimes be a functional strategy in identity exploration. However, in BPD, loneliness is intolerable and a source of suffering and affected individuals tend to seek various means to avoid being alone. This could therefore also explain a decreased exploration in breadth [38].

Based on the averages obtained from the DIDS scores, our control sample is in an identity status of the “identity realization” type and, therefore, the most successful and mature status. Our sample of young adults is in a status called a moratorium. This corresponds to a state of exploration and a lack of engagement. It is a state of transitional crisis at the identity level [27]. DIDS does not have standardized norms, and we should evaluate different identity statuses according to z-scores. Nevertheless, our sample would not be in a state of identity diffusion as predicted by authors such as Luyckx and Luyten [6,27]. Identity diffusion, according to Kernberg, corresponds to a lack of integration of representations of self and others, which can be distorted or fragmented [16]. In a study by Jorgensen in 2009, 66 patients with BPD and 65 control adults were compared. The study did not demonstrate any significant difference between the different identity statuses. Nevertheless, within their sample, Jorgensen estimated that 60% of the patients were in a state of identity diffusion [23]. The reactions of patients with BPD are often unpredictable and under the control of their emotions and impulses. They are often unable to make sense of their actions or understand the behavior that contributes to maintaining an incoherent self and a diffusion of identity [39]. Our sample is a group of participants of transitional age whose identity has not yet been fully established. Moratorium status is a state of crisis and transition that involves active identity exploration. The process of identity development can lead to moments of confusion and discomfort, which can prevent individuals from completing their exploration and cause them to revert to previous states, such as foreclosure or identity diffusion. A longitudinal study would have been interesting to assess whether our young adults succeed in completing their identity exploration process and whether they adopt a mode of identity diffusion, as predicted by the authors. Another hypothesis that could explain this status is that our sample of patients with borderline disorder is a population followed in consultation and supported at the psychotherapeutic level, which allows for a positive evolution of their pathology [40].

Regarding the relationship between identity processes and BPD, our findings revealed that commitment processes tended to have a negative association with BPD, while ruminative exploration showed a positive association with BPD. Consequently, feelings of indecisiveness, persistent contemplation of identity alternatives, and difficulties in making commitments appear to be linked to this personality pathology. A study conducted by Peters in 2017 demonstrated that rumination is correlated with identity disturbance. They suggested that prolonged rumination might interfere with the establishment of a stable sense of identity, potentially contributing to the development of BPD traits [41]. Additionally, exploration in breadth showed a positive correlation with BPD. Individuals with BPD are characterized by emotional dysregulation and seem to struggle in establishing a stable foundation from which they can pursue a meaningful future [42].

Finally, we assessed whether there were links between the different states of identity construction and the dimensions as defined by Zanarini. These dimensions concern affective instability, cognitive disorders (episodes of dissociation or the experimentation of transient psychotic ideas), marked impulsivity, and the instability of identity and relationships with others [33,43]. Correlation analyses reveal significant positive associations between the cognitive dimension and exploration in depth. Typically, individuals with dissociation are expected to report a higher level of identity confusion [20], but our results do not demonstrate this. Contrary to expectations, exploration in depth shows a positive correlation. However, it is important to interpret these findings cautiously, as there is no significant difference between the borderline group and the control group regarding this dimension. Although we did not uncover a significant association with the impulsive dimension, it is worth noting that suicide attempts, which are impulsive behaviors, are part of risky behaviors observed during episodes of strong emotional dysregulation. The total DIDS and the identification with commitment was negatively associated with the number of suicide attempts. Impulse control problems and negative emotionality often lead to difficulties with self-regulation and self-awareness. Becker et al. showed that identity disorders were associated in a sample of adults with DSM criteria such as anger outbursts, impulsivity, and unstable interpersonal relationships, but not with other criteria such as fear of violence, abandonment, suicidal thoughts, and suicide attempts, contrary to our results [44]. In Linehan’s cognitive–behavioral model, the dysregulation of emotions and related impulsive behaviors is closely linked to identity disorders [45].

### Limitations

A number of limitations may affect the interpretation of our results.

One, a small number of young adults with BPD were included in our sample, only 44. This limited the power of the study and did not allow us to conduct a hierarchical cluster analysis, using a method with squared Euclidean distances that would have allowed us to assess different identity status profiles within our sample and assess the links between them and the dimensions of the BPD. Furthermore, in this study, we limited ourselves to uncorrected correlation analyses following this restricted sample size. Our results should therefore be taken with caution. To confirm our results, the study should be replicated with a larger number of individuals with BPD and with more sociodemographic factors like life context or a history of hospitalization. Moreover, we sampled from a student population, which means that our results may not apply to the general population, and we also did not check whether differences existed within the clinical population by place of recruitment (inpatient or outpatient).

Two, in our non-clinical sample, none reported a psychiatric history or any comorbidities, but this was based only on self-report. We also did not exclude individuals with a first-degree family psychiatric history. This introduced bias and should also be taken into account when considering the generalizability of the results. The absence of psychiatric pathologies was not confirmed by a psychiatric assessment by the interviewer.

Three, the DIDS is a self-assessment and BPD patients have mentalization and self-assessment difficulties. It is difficult to provide precise information on the important aspects of their identity [38]. This could explain analyses that turned out to be insignificant. Assessing identity would require a specially designed interview that would make it possible to assess the different identity profiles.

Four, the current study was cross-sectional and thus could only describe identity status at the time of the assessment, i.e., the transition period from adolescence to adulthood. Identity formation is an ongoing process in human development. It would therefore be useful to design longitudinal studies that would make it possible to differentiate a normal identity disturbance from a pathological one over time.

Finally, Luyckx’s model operationalized in the DIDS has proven to be fruitful in developmental research, but not yet in clinical research. The poor internal reliability of the subscale of exploration in depth could also have influenced our results [31].

## 5. Conclusions

In conclusion, this study aimed to evaluate the DIDS scale in a population of young adults with BPD aged 16 to 25 and to examine differences in identity construction within this group. The findings should be understood within a developmental framework, as this age range represents a crucial period of transition to adulthood marked by significant physiological, psychological, and social changes [46,47].

Identity formation in our clinical sample was found to differ from a control population of the same age. Indeed, our analysis of DIDS scores revealed significant differences between young adults with BPD and the control group, particularly in commitment making, exploration in breadth, and identification with commitment. Lower levels of the identity dimension exploration in breadth were related to BPD and not explained by a range of external confounding factors, unlike other dimensions.

Moreover, our findings suggest that ruminative exploration is positively correlated with BPD and commitment processes are negatively correlated, indicating a tendency for individuals with BPD to engage in repetitive exploratory processes and struggle in evaluating alternative identity options.

Furthermore, our study examined the relationship between identity development stages and dimensions defined by Zanarini, including affective instability, cognitive disturbances, and impulsivity in individuals with borderline personality disorder (BPD). We found a positive correlation between cognitive dimension and in-depth exploration but no significant association with impulsivity. However, suicide attempts, considered impulsive behaviors, were negatively associated with overall identity development and commitment. These findings suggest complex interactions between identity, emotion dysregulation, and impulsive behaviors in BPD individuals.

However, several limitations should be noted, including the small sample size, potential biases in self-reported psychiatric history and comorbidities, the use of a self-assessment tool in a population with mentalization and self-assessment difficulties, the cross-sectional design, and the reliability of certain subscales of the DIDS. Future research should address these limitations and consider longitudinal designs to better understand the dynamic nature of identity formation in individuals with BPD.

## Figures and Tables

**Table 1:** Sample description (*n* = 132).

Variables	Categories	%	Controls (*n* = 88)	BPD Individuals (*n* = 44)	*p*-Value Chi^2^	*p*-Value Fisher’s Exact Test
Gender	Male (*n* = 25)	19.0%	25.0%	6.8%	0.012	0.017
	Female (*n* = 107)	81.0%	75.0%	93.2%		
Married	No (*n* = 132)	100%	100%	100%	Not applicable	Not applicable
	Yes (*n* = 0)	0%	0%	0%		
Children	No (*n* = 132)	100%	100.0%	100%	Not applicable	Not applicable
	Yes (*n* = 0)	0%	0.0%	0%		
Living parents	No (*n* = 7)	5.3%	2.3%	11.4%	0.028	0.041
	Yes (*n* = 125)	94.7%	97.7%	88.6%		
Place in the siblings	Single child (*n* = 25)	18.9%	17.0%	22.8%	0.001	0.001
	Eldest child (*n* = 36)	27.3%	21.6%	38.6%		
	Middle child (*n* = 22)	16.7%	12.5%	25.0%		
	Younger child (*n* = 49)	37.1%	48.9%	13.6%		
Student	No (*n* = 14)	10.6%	10.2%	11.4%	0.842	0.999
	Yes (*n* = 118)	89.4%	88.9%	88.6%		
Somatic history	No (*n* = 92)	69.7%	72.7%	63.6%	0.284	0.318
	Yes (*n* = 40)	30.3%	27.3%	36.4%		
Surgical history	No (*n* = 59)	44.7%	39.8%	54.5%	0.108	0.138
	Yes (*n* = 73)	55.3%	60.2%	45.5%		
Psychiatric history	No (*n* = 111)	84.1%	100.0%	52.3%	<0.001	<0.001
	Yes (*n* = 21)	15.9%	0.0%	47.7%		
Psychiatric comorbidities	No (*n* = 105)	79.5%	100.0%	38.6%	<0.001	<0.001
	Yes (*n* = 27)	20.5%	0.0%	61.4%		
Smoking	No (*n* = 111)	84.1%	95.5%	61.4%	<0.001	<0.001
	Yes (*n* = 21)	15.9%	4.5%	38.6%		
Alcohol	No (*n* = 32)	24.2%	27.3%	18.2%	0.043	0.048
	Occasional (*n* = 80)	60.6%	53.4%	75.0%		
	Binge drinking (*n* = 20)	15.2%	19.3%	6.8%		
Drugs	No (*n* = 105)	79.5%	94.3%	50%	<0.001	<0.001
	Yes (*n* = 27)	20.5%	5.7%	50%		
Family psychiatric history	No (*n* = 75)	56.8%	78.4%	13.6%	<0.001	<0.001
	Yes (*n* = 57)	43.2%	21.6%	86.4%		
History of suicide attempts	No (*n* = 88)	66.7%	87.5%	25.0%	<0.001	<0.001
	Yes (*n* = 44)	33.3%	12.5%	75.0%		
History of psychological follow-up	No (*n* = 82)	62.1%	83.0%	20.5%	<0.001	<0.001
	Yes (*n* = 50)	37.9%	17.0%	79.5%		
Psychotropic treatment	No (*n* = 100)	75.8%	100%	27.3%	<0.001	<0.001
	Yes (*n* = 21)	24.2%	0%	72.7%		
Criminal history	No (*n* = 130)	98.5%	98.9%	97.7%	0.614	0.999
	Yes (*n* = 2)	1.5%	1.1%	2.3%		
Educational level	Primary education or no diploma (*n* = 65)	49.3%	38.6%	70.5%	0.001	<0.001
	Secondary education (*n* = 39)	29.5%	31.8%	25.0%		
	Higher education (*n* = 28)	21.2%	29.6%	4.5%		
	Median (P25–P75)				Wilcoxon test	
Age (years)	18.0 (17.0–22.0)		18.0 (17.0–22.0)	18.0 (16.0–21.0)	0.051	
Number in siblings	1.0 (1.0–2.0)		1.0 (1.0–2.0)	1.0 (1.0–2.0)	0.304	
Number of suicide attempts	0.0 (0.0–1.0)		0.0 (0.0–0.0)	2.0 (0.0–5.0)	<0.001	
DIDS commitment making	19.0 (15.0–23.0)		21.0 (18.0–24.0)	16.0 (10.0–20.0)	<0.001	
DIDS exploration in breadth	20.0 (17.0–22.0)		21.0 (19.0–23.0)	17.0 (13.0–20.0)	<0.001	
DIDS ruminative exploration	16.0 (11.0–20.0)		14.0 (10.0–19.0)	17.0 (13.0–22.0)	0.044	
DIDS identification with commitment	17.0 (14.0–21.0)		19.0 (16.0–23.0)	12.0 (8.0–17.0)	<0.001	
DIDS exploration in depth	17.0 (15.0–20.0)		18.0 (15.0–20.0)	17.0 (14.0–20.0)	0.224	
DIDS total	90.0 (82.0–97.0)		92.0 (86.0–99.0)	81.0 (63.0–93.0)	<0.001	
Number of BPD criteria (DSM 5)	0.0 (0.0–5.0)		0.0 (0.0–0.0)	6.0 (5.0–7.0)	<0.001	
Affective dimension			/	9.0 (8.0–10.0)		
Cognitive dimension			/	3.0 (2.0–4.0)		
Impulsive dimension			/	7.0 (5.0–8.0)		
Interpersonal dimension			/	10.0 (8.0–12.0)		

**Table 2:** Adjusted results for DIDS (*n* = 132).

Variables	b_a1_ (ES) Controls vs. BPD Individuals
DIDS commitment making	1.2 (2.6)
DIDS exploration in breadth	−3.9 (1.8) *
DIDS ruminative exploration	−4.8 (3.4)
DIDS identification with commitment	−2.0 (2.7)
DIDS exploration in depth	−0.2 (2.3)
DIDS total	−4.0 (7.1)

b_a1_ (ES): Quantile regression coefficient adjusted (standard error). These coefficients are the difference of median between controls and BPD individuals, adjusted for gender, living parents, place in the siblings, psychiatric history, psychiatric comorbidities, smoking, alcohol, drugs, family psychiatric history, history of suicide attempts, history of psychological follow-up, psychotropic treatment, and educational level. * *p* < 0.05.

**Table 3:** Correlation between BPD features and DIDS in whole sample (*n* = 132).

Variables	BPD
DIDS commitment making	−0.395 *
DIDS exploration in breadth	−0.467 *
DIDS ruminative exploration	0.177 *
DIDS identification with commitment	−0.511 *
DIDS exploration in depth	−0.107
DIDS total	−0.425 *

* *p* < 0.05.

**Table 4:** Correlation between BPD dimensions and DIDS in BPD individuals (*n* = 44).

	Affective Dimension	Cognitive Dimension	Impulsive Dimension	Interpersonal Dimension	Number of SA
DIDS commitment making	−0.060	0.135	−0.125	0.048	−0.163
DIDS exploration in breadth	−0.016	0.200	−0.104	0.159	−0.192
DIDS ruminative exploration	0.095	0.123	−0.070	−0.002	0.064
DIDS identification with commitment	0.027	0.093	−0.069	0.107	−0.380 *
DIDS exploration in depth	0.152	0.300 *	−0.240	−0.027	−0.128
DIDS total	0.016	0.186	−0.232	0.065	−0.351 *

SA = Suicide attempt. * *p* < 0.05.

## Data Availability

The corresponding author may provide the data for this study upon reasonable request.
